# Effects of Oral Iron Supplementation on Blood Iron Status in Athletes: A Systematic Review, Meta-Analysis and Meta-Regression of Randomized Controlled Trials

**DOI:** 10.1007/s40279-024-01992-8

**Published:** 2024-02-26

**Authors:** Anja Neža Šmid, Petra Golja, Vedran Hadžić, Ensar Abazović, Kristina Drole, Armin H. Paravlic

**Affiliations:** 1 Biotechnical Faculty, University of Ljubljana, Jamnikarjeva ulica 101, 1000 Ljubljana, Slovenia; 2 Faculty of Sport, University of Ljubljana, Gortanova ulica 22, 1000 Ljubljana, Slovenia; 3 Faculty of Sport and Physical Education, University of Sarajevo, Patriotske Lige 41, 71000 Sarajevo, Bosnia and Herzegovina; 4 Faculty of Sports Studies, Masaryk University, 625 00 Brno, Czech Republic

## Abstract

**Background:**

Iron deficiency in athletes is initially treated with a nutritional intervention. If negative iron balance persists, oral iron supplementation (OIS) can be used. Despite the recent proposal for a refinement of treatment strategies for iron-deficient athletes, there is no general consensus regarding the actual efficiency, dosage, or optimal regimen of OIS.

**Objective:**

The aim of this meta-analysis was to evaluate to what extent OIS affects blood iron parameters and physical performance in healthy adult athletes.

**Methods:**

PubMed, Web of Science, PEDro, CINAHL, SPORTDiscus, and Cochrane were searched from inception to 2 November 2022. Articles were eligible if they satisfied the following criteria: recruited subjects were healthy, adult and physically active individuals, who used exclusively OIS, irrespective of sex and sports discipline. Exclusion criteria: simultaneous supplementation with iron and any other micronutrient(s), intravenous iron supplementation or recent exposure to altitude acclimatisation. The methodological quality of included studies was assessed with the PEDro scale, the completeness of intervention reporting with the TIDieR scale, while the GRADE scale was used for quality of evidence synthesis. The present study was prospectively registered in PROSPERO online registry (ID: CRD42022330230).

**Results:**

From 638 articles identified through the search, 13 studies (*n* = 449) were included in the quantitative synthesis. When compared to the control group, the results demonstrated that OIS increases serum ferritin (standardized mean difference (SMD) = 1.27, 95% CI 0.44–2.10, *p* = 0.006), whereas blood haemoglobin (SMD = 1.31, 95% CI − 0.29 to 2.93, *p* = 0.099), serum transferrin receptor concentration (SMD = − 0.74, 95% CI − 1.89 to 0.41, *p* = 0.133), and transferrin saturation (SMD = 0.69, 95% CI − 0.84 to 2.22, *p* = 0.330) remained unaltered. Following OIS, a trend of small positive effect on *V*O_2max_ (SMD = 0.49, 95% CI − 0.09 to 1.07, *p* = 0.086) was observed in young healthy athletes. The quality of evidence for all outcomes ranged from moderate to low.

**Conclusions:**

Increase in serum ferritin concentration after OIS was evident in subjects with initial pre-supplementation serum ferritin concentration ≤ 12 µg/l, while only minimal, if any effect, was observed in subjects with higher pre-supplementation serum ferritin concentration. The doses of OIS, that induced a beneficial effect on hematological parameters differed from 16 to 100 mg of elementary iron daily, over the period between 6 and 8 weeks. Shorter supplementation protocols have been shown to be ineffective.

**Supplementary Information:**

The online version contains supplementary material available at 10.1007/s40279-024-01992-8.

## Key Points

**Table:** 

Our meta-analysis revealed that oral iron supplementation (OIS) increases serum ferritin concentrations in healthy adult athletes.
The effects of OIS were shown to be greater in subjects with initially lower serum ferritin concentration i.e., ≤ 12 µg/l, while only minimal, if any, effect was observed in subjects with higher pre-supplementation serum ferritin concentration.
A duration of more than 6 weeks of OIS should be applied to induce greater benefits in serum ferritin concentrations.
OIS showed no beneficial effects on improving maximal aerobic capacity of healthy adult athletes.

## Introduction

The term ‘iron deficiency’ includes a plethora of physiological deficits induced by negative iron balance [1]. The early phase of iron deficiency is known as iron deficiency non-anaemia (IDNA) during which the haemoglobin concentration remains normal, but serum ferritin is decreased [2]. It is worth noting that important sex differences in normal haemoglobin concentrations exist, so what is considered normal in females (reference values: 115–165 g/l) may not be considered normal in males (reference values 125–185 g/l) [3]. Although there is no consensus [4] on a serum ferritin concentration that indicates iron deficiency with haemoglobin concentration above 115 g/l [5], values below 35 µg/l of serum ferritin have been suggested for iron depletion, and values below 20 µg/l for a limited supply of iron to bone marrow and thus the first reduction in haemopoiesis [5]. If IDNA is left untreated, it can progress to iron deficiency anaemia [2], characterized by a decrease in haemoglobin concentration below 115 g/l [5] and serum ferritin concentration below 12 µg/l [5]. In cases of chronic inflammatory conditions, serum ferritin concentration as high as 100 μg/l or serum transferrin saturation of less than 20% are considered diagnostic for iron deficiency [6].

Iron deficiency is common in physically active individuals and particularly exacerbated in professional athletes [7, 8]. Athletes are prone to iron loss due to prolonged physical activity, which puts high physiological demands on their bodies. Apart from menstrual iron loss in women, high physical and environmental stress can increase sweating rate, induce gastrointestinal bleeding, and cause haemolysis due to mechanical forces, hematuria, thermohaemolysis, and lower iron recycling rates due to inflammation [9, 10]. Elite athletes, engaged in extremely physiologically demanding sports like cycling, marathon running, or triathlon, commonly exhibit decreased haemoglobin concentrations [11]. However, when explaining the latter, one needs to be careful, as low haemoglobin concentration may reflect dilutional pseudoanaemia resulting from plasma volume expansion in endurance athletes, or may in fact indicate a greater risk of developing iron deficiency anaemia compared to sedentary individuals due to the factors presented above [12].

The prevalence of iron deficiency in athletes is reported to be between 3 and 11% in males and at 15–35% in females [13]. Female athletes engaged in endurance, aesthetic, or weight class sports are at an inherently higher risk of developing iron deficiency [14], due to their dietary intake, which is often energy deficient and as such insufficient for their needs [15]. Iron is an indispensable constituent of haemoglobin and myoglobin, as well as of several enzymes of the respiratory chain and energy metabolism, involved in more than 180 biochemical reactions in humans [16]. It is also involved in blood pH buffering [17]. Thus, it is not surprising that insufficient iron status, starting with iron deficiency non-anaemia, is associated with fatigue, which may present as lack of energy, tiredness, decreased work and training capacity, performance impairment, poorer competition results, impaired muscle function, and impaired stress management [4, 18, 19].

Nearly 90% of daily iron needs are obtained from the breakdown of erythrocytes [20]. To prevent iron deficiency, the remainder must be replenished by diet [20]. The initial approach when an athlete is faced with iron deficiency is a nutritional intervention [2]. However, if negative iron balance persists even after the nutritional intervention, the next step is usually oral iron supplementation (OIS) [21]. The latter can be performed with the use of different iron salts, with iron sulphate being the most commonly used. However, the bioavailability (i.e. the proportion of ingested substance that enters the bloodstream) of iron is low, as iron balance is regulated by its absorption and no physiological mechanism exists to regulate its secretion [20]. This makes it challenging for athletes to meet their iron needs. Furthermore, hepcidin, a hormone released by hepatocytes, decreases iron absorption in enterocytes when levels of iron storing intracellular protein ferritin are high, and its levels increase during inflammation [22], which is a common consequence of endurance training [23]. Thus, it seems reasonable to expect that regular endurance training can decrease iron absorption. It has also been reported that higher dosages of iron intake result in diminished fractional iron absorption within the 24 h following ingestion [24–26], although the absolute absorption may actually be enhanced. This is most likely due to an increase in hepcidin levels immediately after supplementation, which presents an obstacle to replenishment of iron stores. Last but not least, OIS can cause significant gastrointestinal irritation [27], which makes it even more difficult for athletes to adhere to supplementation. In more serious cases of iron deficiency, when a rapid and efficient approach is required, intramuscular or intravenous iron administration has been recommended [13]; however, this is also associated with some risks of adverse reactions.

All of the aforementioned factors can affect the efficacy of iron supplementation in athletes. Although a refinement of treatment strategies for iron deficient athletes has recently been proposed [2], there is still no general consensus in the scientific literature regarding the optimal dosage, regimen, or overall effectiveness of dietary iron supplementation for improving iron status among athletes. For example, DellaValle and Haas [18] found no significant changes in any iron status indices after 6 weeks of iron supplementation with 20 mg of elementary iron/day (i.e. 100 mg of iron sulphate/day; 50 mg twice daily) in non-anaemic highly trained [28] female rowers with an average initial iron serum ferritin concentration of 28 µg/L. In contrast, using higher supplementation doses for 6 weeks (30 mg of elemental iron/day; i.e. 150 mg ferrous sulphate/day), Hinton and Sinclair [29] reported that serum ferritin considerably increased (by 77%) in trained [28] men and women with an average initial iron serum ferritin concentration of 12 µg/L, but only reached the lower normal concentration (21 µg/L) after supplementation. Furthermore, very high doses of supplemental iron (130 mg elementary iron/day; i.e. 650 mg of iron sulphate/day) did not affect blood iron or performance parameters in highly trained [28] female runners with baseline ferritin concentration within the normal range (average initial iron serum ferritin concentrations of 29 µg/L) [30]. Powell and Tucker [30] therefore suggested that OIS provides no beneficial effects in athletes with baseline ferritin concentration within the normal range (while proposing the normal range of serum ferritin between 12 and 135 µg/l), but recapitulated that OIS beneficially affects serum ferritin by increasing it to within normal levels in those athletes with initially low (below 12 µg/l) ferritin concentration.

A meta-analysis by Burden et al. [31] investigated the effects of iron treatment on iron status in iron deficient but non-anaemic endurance athletes and demonstrated that iron treatment indeed improves iron stores and blood iron parameters. However, their meta-analysis combined studies that applied both oral and intramuscular iron injection treatment, with the later exerting the highest effects on serum ferritin levels. The efficiency of OIS *alone* for iron balance remains to be determined. Therefore, this systematic review and meta-analysis aimed to investigate whether and to what extent OIS affects blood iron parameters in healthy adult athletes.

## Methods

### Population

Only published, peer-reviewed, randomized controlled studies accessible in full text and published in English or Serbian language, investigating OIS in healthy, adult (≥ 16 years), physically active individuals/athletes, who were at least “Tier 1: recreationally active”[28], were included in the literature review and meta-analysis. Studies were included in the literature review if their subjects exclusively used OIS, irrespective of their sex and sports discipline. Studies were included in the literature review if the control group received either placebo treatment or no treatment at all.

### Intervention

The effects of exclusively OIS on haematological (blood haemoglobin concentration, serum ferritin concentration, serum transferrin receptor concentration, transferrin saturation) and performance-related parameters (maximal oxygen consumption [*V*O_2max_] were examined. Studies were not included in the literature review if (a) subjects used simultaneous supplementation with iron and any other micronutrient(s), as in such cases the effects of individual micronutrient supplementation cannot be discerned from others; the only exception made was when subjects used oral vitamin C supplementation together with oral iron supplementation—in such cases, the study was included in our analysis, (b) intravenous iron supplementation was used, as the aim of the study was to investigate the effects of oral and not of any other form of iron supplementation; and (c) the subjects had recent exposure to altitude acclimatisation, as this would have affected haematological parameters per se.

This review was planned and performed in accordance with the Preferred Reporting Items for Systematic Reviews and Meta-Analyses (PRISMA 2020) [32]. The following six electronic bibliographic databases were searched for relevant studies: PubMed, Web of Science, PEDro, CINAHL, SPORTDiscus, and Cochrane. The combination of terms “iron AND athletes AND supplementation” was used in the search engines of all databases. The last search was performed on 2 November 2022.

Criteria for data extraction were discussed and accepted by the team members. Data extraction according to the pre-set criteria was undertaken by one team member and any uncertainties were discussed with other team members until agreement was reached.

**Fig. 1:**
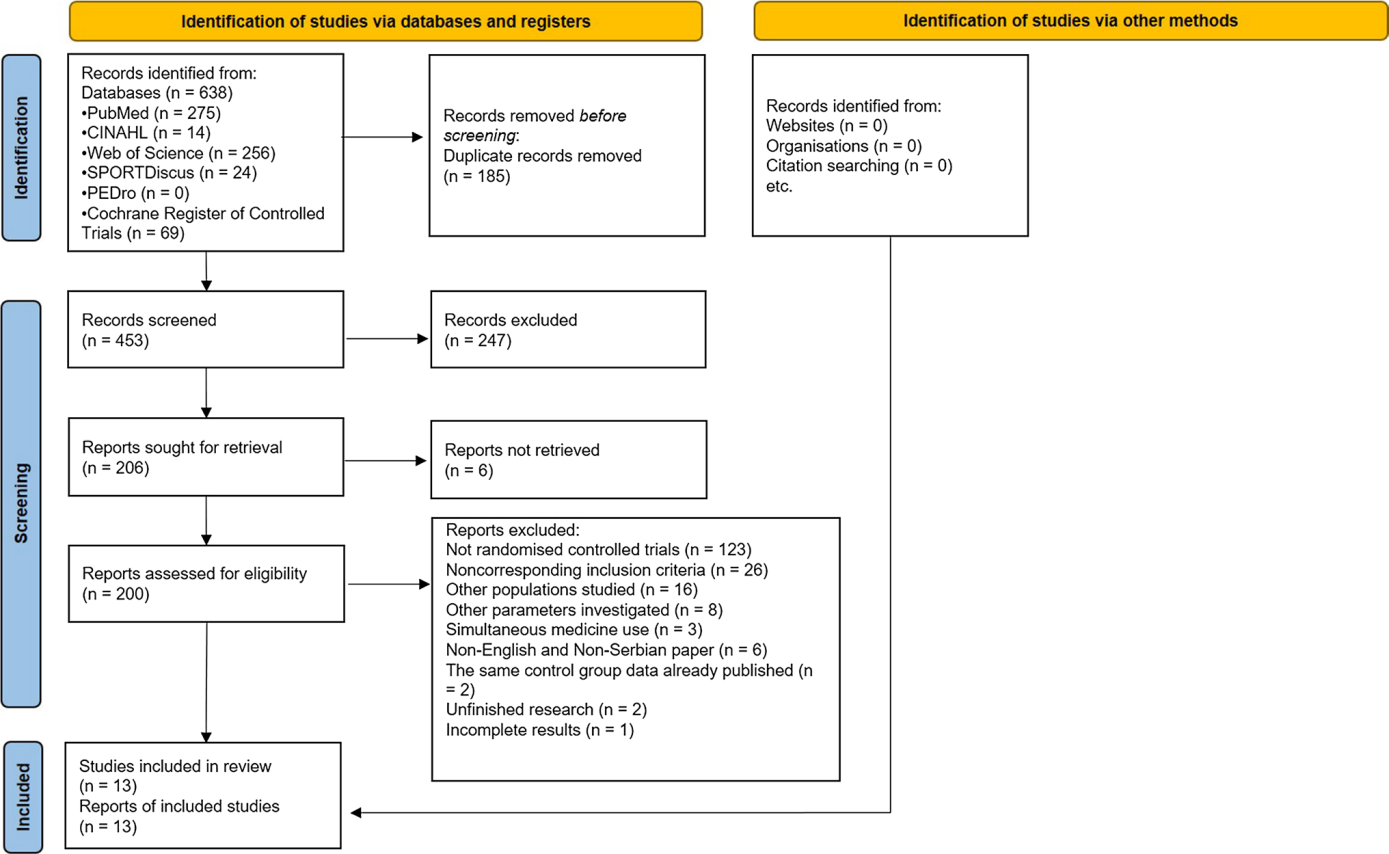
PRISMA flow diagram of study selection process

The process of eligible study selection is presented in Fig. 1.

A detailed list of papers identified at each step of the study selection process is available in Online Supplementary Material 1.

### Comparison

The selected haematological and performance-related parameters were compared between the control (i.e. placebo) and experimental (i.e. iron supplemented) group, as well as within groups (i.e. pre- and post-supplementation).

### Outcomes

Pre- and post-supplementation data on haematological (blood haemoglobin concentration, serum ferritin concentration, serum transferrin receptor concentration, transferrin saturation, and performance-related parameters (maximal oxygen consumption, *V*O_2max_) were studied. Data on sex, age, sports discipline, daily iron supplementation dose, form of ingested iron, and duration of supplementation were also extracted from the eligible studies.

### Quality Assessment

The methodological quality of the included studies was assessed using the Physiotherapy Evidence Database (PEDro) scale [33], with a PEDro score below three denoting poor quality, a score of 4–5 denoting fair quality, and a score of 6–10 denoting high quality studies [34].

The completeness of intervention reporting was assessed with the Template for Intervention Description and Replication (TIDieR) scale [35].

The Grading of Recommendations, Assessment, Development, and Evaluations (GRADE) was used to evaluate the quality of evidence for each study included in the present meta-analysis. For each potential risk factor, including *design limitations* (PEDro score of included studies < 6), *imprecision* (less than 300 participants for each pooled outcome measure), and *inconsistency* (moderate to high heterogeneity; *I*^2^ ≥ 50%), the level of certainty was adopted as high (when considerable confidence existed that the true effect was similar to the estimated effect), moderate (when the true effect was probably close to the estimated effect), low (when the true effect might have been markedly different from the estimated effect), or very low (when the true effect was probably markedly different from the estimated effect). The inclusion criteria guaranteed a specified population with relevant outcomes, and hence the *indirectness* criterion was not accounted for when rating the quality of the evidence in this review [36].

### Study Registration

The present study was performed according to the guidelines of the International Prospective Register for Systematic Reviews (PROSPERO) and was registered in the PROSPERO database with ID = CRD42022330230.

### Statistical Analysis

Statistical analysis was performed with SPSS statistical software (version 29.0, IBM Inc, Chicago, IL, United States of America). Due to the large heterogeneity observed for each meta-analysis, data were analysed using a random effect model. The method of restricted maximum likelihood (REML) with Knap-Hartung standard error adjustment was used for all analyses [37]. Egger’s test was performed on collected data to provide statistical evidence of publication bias. Given the variability of methods used to analyse blood samples and the variability of units reported, a standardized mean difference (SMD) along with 95% confidence intervals (CIs) were calculated for all outcome measures. SMD was set as trivial (< 0.20), small (0.21–0.60), moderate (0.61–1.20), large (1.21–2.00), very large (2.01–4.00), or extremely large (> 4.00) [38]. Heterogeneity was assessed using the I^2^ statistic that indicates the percentage of variability across studies due to heterogeneity rather than chance. Values of 25%, 50% and 75% represent low, moderate and high heterogeneity, respectively [39]. Additionally, sensitivity analysis was performed by eliminating the study with the largest effect from the analysis. Moreover, to investigate the potential moderators of effects of iron supplementation on variables of interest, several sub-group meta-analyses were performed by comparing groups with: (a) initially lower (≤ 12 μg/l) vs. higher (> 12 μg/l) serum ferritin concentration; (b) shorter (< 6 weeks) vs. longer (≥ 6 weeks) intervention period; (c) lower doses of average elemental iron intake (< 60 mg/day) vs. higher doses (≥ 60 mg/day); and (d) initially lower (< 134 g/l) vs. higher (≥ 134 g/l) haemoglobin levels. The cut-off values for serum ferritin concentrations were determined based on our literature review [5, 30, 40]. Other cut-off values were established using average values reported in the original studies.

Furthermore, random-effects meta-regression using the REML was performed to examine whether the effects of OIS on haemoglobin and serum ferritin concentration were moderated by initial ferritin concentration, average daily dose of elemental iron intake, age of participants, duration of supplementation, and the methodological quality of included studies (PEDro score). To minimize the risk of overfitting, a meta-regression was performed when a minimum of 10 studies were eligible per examined covariate [41]. A level of *p* < 0.05 was adopted as statistically significant.

## Results

### Literature Review

#### Study Selection

The search “iron AND athletes AND supplementation” performed in 5 databases (PubMed, CINAHL, Web of Science, SPORTDiscus, PEDro) and 1 register (Cochrane Register of Controlled Trials) yielded 638 papers, of which 185 duplicate papers were excluded. Papers (*n* = 453) were then screened and 247 papers excluded, as their topic was unrelated to our study. Of the remaining 206 papers 6 were not retrieved, meaning 200 papers were then assessed for eligibility and 187 papers were excluded for one or more of the following reasons: (a) the paper was not a randomised controlled trial (*n* = 123), (b) noncorresponding inclusion criteria were used (*n* = 26), (c) populations other than adult healthy physically active individuals were studied (*n* = 16), (d) parameters other than those we were interested in were investigated (*n* = 8), (e) not only OIS, but also other medicine/supplementation was simultaneously used (*n* = 3), (f) the paper was not published in English or Serbian (*n* = 6), (g) the same control group data had already been published (*n* = 2), (h) unfinished research (*n* = 2) and (i) incomplete results (*n* = 1).

#### Study Characteristics

Each of the 13 studies included in the meta-analysis was a randomised controlled trial, which included a control and an experimental (i.e. OIS) group. The experimental group received OIS and no other form of iron or other micronutrient supplementation for the period from 3 days to 8 weeks. In 12 studies, the control group received placebo treatment over the same period of time as the control group, while in one study [42] the control group received no treatment. In 10 out of 13 studies, iron was supplemented in a form of ferrous sulfate provided in capsules or pills, while in two studies [22, 43], iron was provided in a solution. Subjects ingested iron supplements once or twice daily. Across the studies, doses of supplemental elemental iron varied between 15 and 100 mg/day.

#### Participants’ Characteristics

A total of 449 adult, physically active individuals/athletes participated in the studies, consisting of 432 females and 17 males. There were 224 subjects in the experimental and 225 subjects in the control group, making 449 subjects altogether, as 10 females served in both the experimental and control groups in the study by Powell and Tucker [30]. Participants’ age was between 18 and 28 years, with the exception of one study [42], in which the youngest subjects were 16 years old.

A systematic overview of the studies included in the meta-analysis with their main characteristics and outcomes is presented in Table 1. The additional information about the study interventions were provided in Supplementary Table 1.

**Table 1:** Main characteristics of the studies included in the meta-analysis

Study	Sex (F/M)	N (exp/ncontrol)	Age exp (years; AVG ± SD)	Age control (years; AVG ± SD)	Training calibre of athletes (according to McKay et al., 2020 [28])	Dose of elemental iron supplemented (mg/day)	Form of iron supplement	Dose of elemental iron (mg/day)	Frequency of supplementation	Duration of supplementation	Intervention in the control group	Exercise load during supplementation	Parameter studied (main outcome)
Ishibashi et al. 2017 [22]	0/14	7/7	20.5	20.5	Tier 3: highly trained	24	Iron in a flavoured drink	120^a^	Twice daily	3 days	Flavoured drink	Yes	3, 4
DellaValle and Haas 2014 [44]	31/0	15/16	19.7 ± 0.9	19.8 ± 1.1	Tier 3: highly trained	15,8	Ferrous sulfate	100	Twice daily	6 weeks	Placebo capsule with lactosis	Yes	1, 2, 4, 5
Radjen et al. 2011 [42]	17/0	9/8	20.5	20.5	Tier 3: highly trained	40	Ferrous sulfate	200^a^	Once daily	8 weeks	/	No	1, 3, 4, 5
McClung et al. 2009 [45]	165/0	83/82	20.4 ± 4.2	20.8 ± 4.4	Tier 2: Trained/Developmental	15	Ferrous sulfate	100	Once daily	8 weeks	Placebo capsule	Yes	1, 2, 3, 4
Hinton and Sinclair 2007 [29]	17 / 3	10/10	28.1 ± 5.1	27.7 ± 4.4	Tier 2: trained	30	Ferrous sulfate	150^a^	Once daily	6 weeks	Placebo capsule with lactosis	Yes	1, 2, 3, 4, 5
Kang and Matsuo 2004 [43]	25/0	11/14	22.6 ± 2	23.8 ± 2.8	Tier 4: Elite/International Level	40	Iron in solution	200^a^	Once daily	4 weeks	Placebo solution	No	1, 4
Hinton et al. 2000 [46]	42/0	22/20	21.0 ± 0.8	20.0 ± 0.4	Tier 2: Trained/Developmental	20 ^b^	Ferrous sulfate	100	Twice daily	6 weeks	Placebo capsule	No	1, 2, 3, 4, 5
LaManca and Haymes 1993 [47]	20/0	10/10	28 ± 2	28 ± 2	Tier 2: Trained/Developmental	100	Ferrous sulfate	318	Twice daily	8 weeks	Placebo capsule	No	1, 3, 4, 5
Klingshirn et al. 1992 [48]	18/0	9/9	29.38 ± 5.76	28.44 ± 3.68	Tier 2: Trained/Developmental	100	Ferrous sulfate	320	Twice daily	8 weeks	Placebo capsule	No	1, 3, 4, 5
Powell and Tucker 1991 [30]	10/0	10/same 10	20.2 ± 1.3	20.2 ± 1.3	Tier 3: highly trained	130	Ferrous sulfate	650	Once daily	2 weeks	Placebo capsule with lactosis	No	1, 3, 4, 5
Magazanik et al. 1991 [49]	28/0	13/15	19	19	Tier 1: Recreationally Active	32	Ferrous sulfate	160	Once daily	6 weeks	Placebo pill	Yes	1, 4, 5
Yoshida et al. 1990 [50]	12/0	6/6	19.7 ± 1.1	19.5 ± 1.1	Tier 3: highly trained	60	Ferrous citrate sodium succinate	200	Three times a day	8 weeks	Matching placebo pill	No	1, 4, 5
Newhouse et al. 1989 [58]	37/0	19/18	28 ± 2	28 ± 2	Tier 2: Trained/Developmental	100	Ferrous sulfate	320	Twice daily	8 weeks	Placebo pill	No	1, 3, 4, 5

*F* females, *M* males, *exp* experimental group, 1—haemoglobin, 2—serum transferrin receptor, 3—transferrin saturation, 4—ferritin, 5—*V*O_2__max_

^a^Values not given in the paper, but calculated as elemental iron: ferrous sulfate = 1:5

^b^Supplementation most likely the same (i.e. 16 mg/day) as in the paper by Brownlie et al., 2004 [39], as haematological data were identical in the two studies

#### Publication Bias Assessment

The results of Egger’s test indicated publication bias for the meta-analysis summarizing results on haemoglobin (*p* = 0.045) only, whereas meta-analyses for ferritin (*p* = 0.101), serum transferrin receptor (*p* = 0.254), transferrin saturation (*p* = 0.645), *V*O_2max_ (*p* = 0.929) and maximum heart rate (*p* = 0.370) were unbiased. The publication bias was considered at *p* < 0.10.

#### Quality Assessment

One study included in the present literature review and meta-analysis obtained a PEDro score of 6 [30], one a PEDro score of 8 [43], one a PEDro score of 9 [22], and the other nine studies a PEDro score of 10 [29, 44–50] (Table 2). Thus, all studies included in the meta-analysis were denoted as high-quality studies according to their PEDro score.

**Table 2:** Fulfillment of Physiotherapy Evidence Database (PEDro) criteria for each of the studies included in the present meta-analysis

Study	PEDro criteria											PEDro score (out of 11)
	1	2	3	4	5	6	7	8	9	10	11	
Ishibashi et al. 2017 [22]	Y	Y	Y	Y	N	N	Y	Y	Y	Y	Y	9
DellaValle and Haas 2014 [44]	Y	Y	Y	Y	Y	N	Y	Y	Y	Y	Y	10
Radjen et al. 2011 [42]	Y	Y	N	Y	N	N	N	Y	Y	Y	Y	7
McClung et al. 2009 [45]	Y	Y	Y	Y	Y	N	Y	Y	Y	Y	Y	10
Hinton and Sinclair 2007 [29]	Y	Y	Y	Y	Y	N	Y	Y	Y	Y	Y	10
Kang and Matsuo 2004 [43]	Y	Y	Y	Y	N	N	N	Y	Y	Y	Y	8
Hinton et al. 2000 [46]	Y	Y	Y	Y	Y	N	Y	Y	Y	Y	Y	10
LaManca and Haymes 1993 [47]	Y	Y	Y	Y	Y	N	Y	Y	Y	Y	Y	10
Klingshirn et al. 1992 [48]	Y	Y	Y	Y	Y	N	Y	Y	Y	Y	Y	10
Powell and Tucker 1991 [30]	N	Y	Y	Y	Y	N	N	Y	N	Y	Y	6
Magazanik et al. 1991 [49]	Y	Y	Y	Y	Y	N	Y	Y	Y	Y	Y	10
Yoshida et al. 1990 [50]	N	Y	N	Y	Y	Y	N	Y	Y	Y	Y	8
Newhouse et al. 1989 [58]	Y	Y	Y	Y	Y	N	Y	Y	Y	Y	Y	10

PEDro criteria: 1—eligibility criteria were specified, 2—subjects were randomly allocated to groups (in a crossover study, subjects were randomly allocated an order in which treatments were received), 3—allocation was concealed, 4—the groups were similar at baseline regarding the most important prognostic indicators, 5—there was blinding of all subjects, 6—there was blinding of all therapists who administered the therapy, 7—there was blinding of all assessors who measured at least one key outcome, 8—measures of at least one key outcome were obtained from more than 85% of the subjects initially allocated to groups, 9—all subjects for whom outcome measures were available received the treatment or control condition as allocated or, where this was not the case, data for at least one key outcome were analysed by “intention to treat”, 10—the results of between-group statistical comparisons were reported for at least one key outcome, 11—the study provided both point measures and measures of variability for at least one key outcome. Y—criterion fulfilled, N—criterion not fulfilled

#### Completeness of Intervention Reporting

**Fig. 2:**
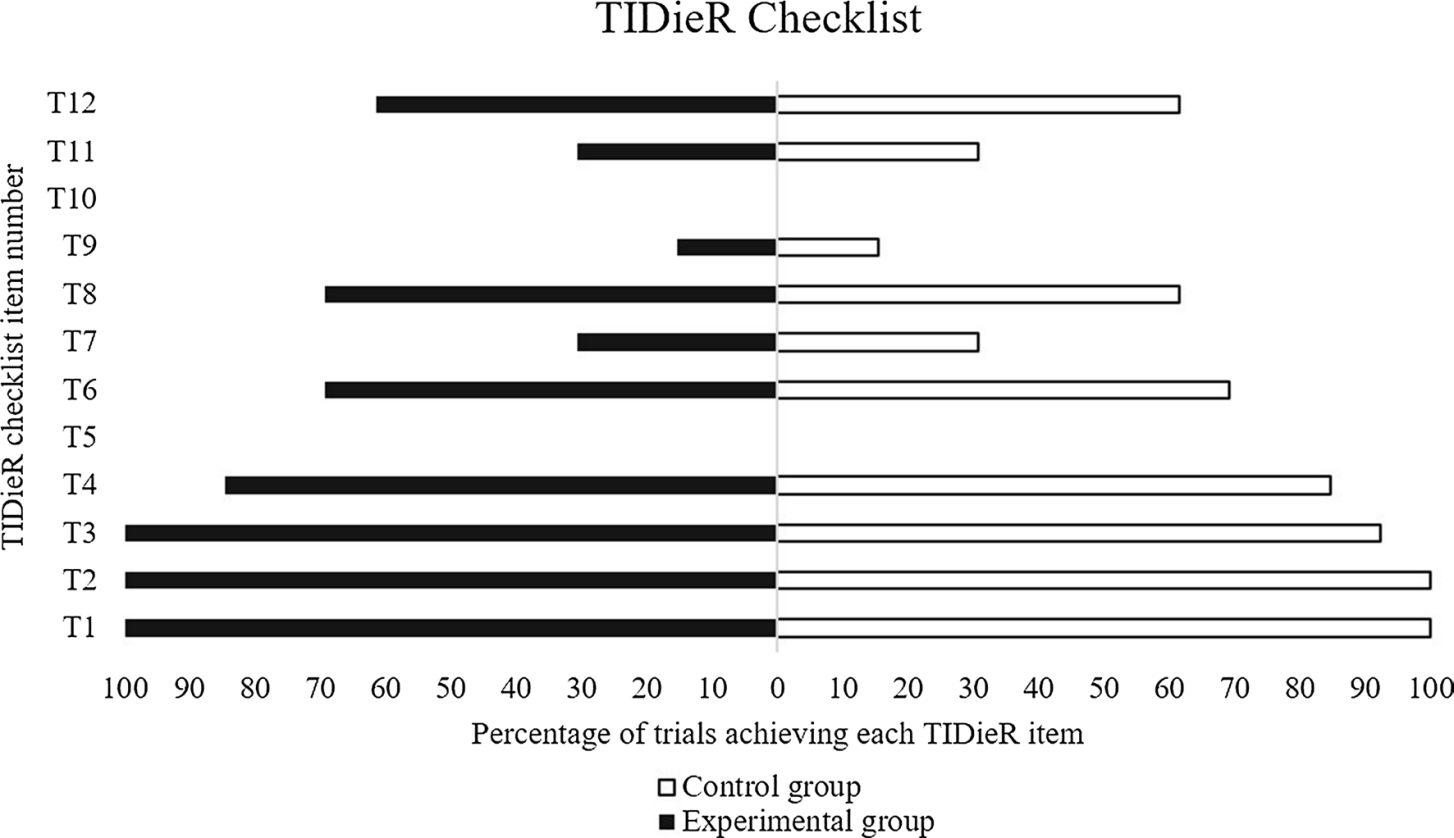
The extent of TIDieR criteria fulfillment (expressed in percentage relative to 12 criteria (T1–T12) possible) for studies included in the present literature review and meta-analysis

Figure 2 presents how many criteria of the TIDieR scale (out of 12) had been fulfilled by each study included in the present literature review and meta-analysis. None of the studies fulfilled all TIDieR criteria.

#### The Grading of Recommendations, Assessment, Development, and Evaluations (GRADE)

The results of Grading of Recommendations, Assessment, Development, and Evaluations (GRADE) are summarised in Table 3.

**Table 3:** Grading of Recommendation, Assessment, Development and Evaluations (GRADE) for results summarized

Outcome	Trials (*n*)	Participants (*n*)	SMD	LLCI	HLCI	*I*^2^	PEDro score	Quality of evidence (GRADE)
Ferritin	13	449	1.27	0.44	2.10	90	9.1	Moderate quality
Blood Hb	12	435	1.31	− 0.29	2.92	97	9.2	Moderate quality
sTRC	4	258	− 0.74	− 1.89	0.41	81	10.0	Low quality
Tsat	9	343	0.69	− 0.84	2.22	95	9.1	Moderate quality
*V*O_2max_	10	243	0.49	− 0.09	1.07	70	9.1	Low quality

*SMD* standardized mean difference, *LLCI* lower limit confidence interval, *HLCI* higher limit confidence interval, *I*^*2*^ heterogeneity measure, *Hb* haemoglobin, *sTRC* serum transferrin receptor concentration, *Tsat* transferrin saturation, *VO*_*2max*_ maximal oxygen consumption

### Meta-Analysis

#### Effects of Iron Supplementation on Iron Status

Supplementary Table 2 presents the results of iron supplementation on concentration of serum ferritin, haemoglobin, serum transferrin receptor, and transferrin saturation. The magnitude and direction of effects, as well as 95% confidence intervals, are presented for both individual studies and summary estimates.

**Fig. 3:**
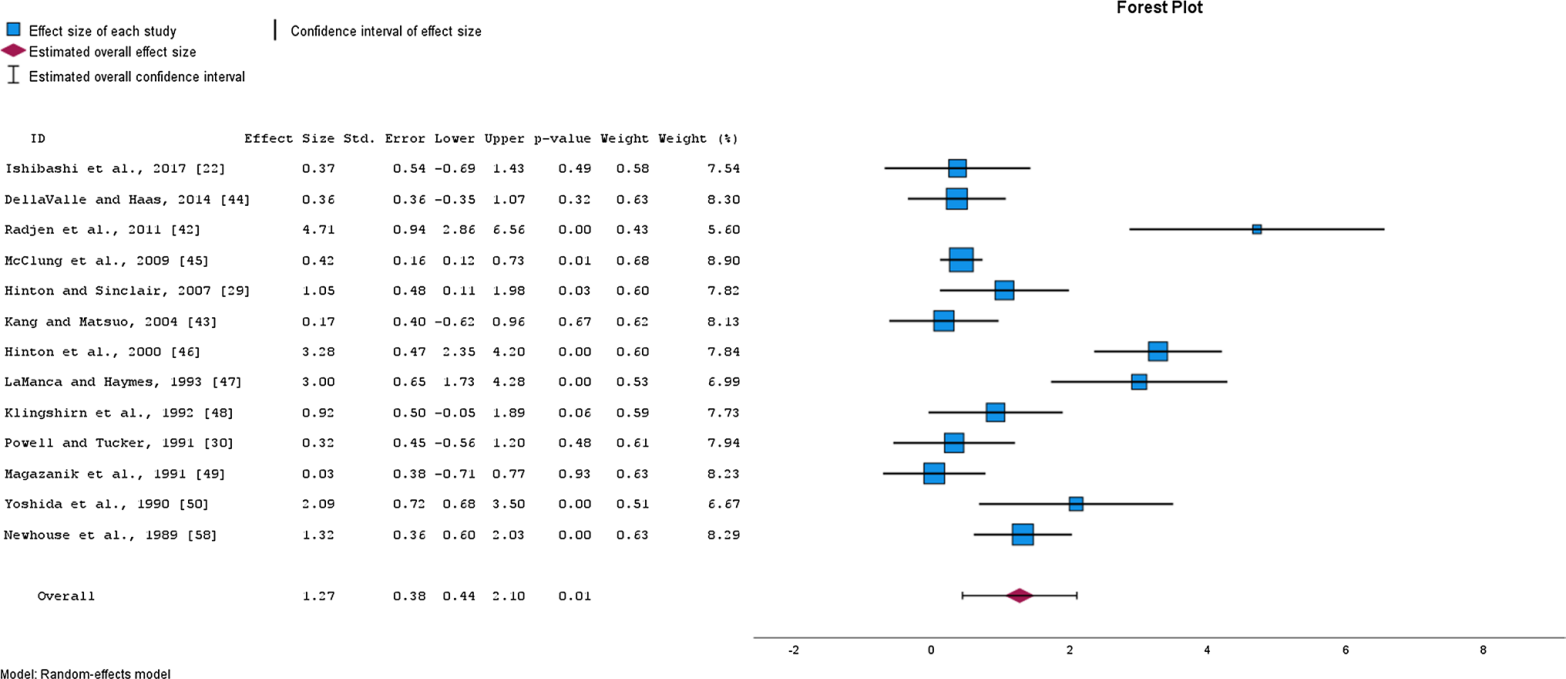
Changes in serum ferritin concentration after oral iron supplementation

Meta-analysis of 13 studies with a total of 449 participants demonstrated a highly significant *large* increase in serum ferritin concentration after OIS (SMD = 1.27, 95% CI 0.44–2.10, *p* = 0.006, *I*^2^ = 90; EXP PRE mean ± SD = 20.4 ± 10.9, EXP POST = 30.3 ± 12.4; CON PRE = 20.0 ± 10.8; CON POST = 21.5 ± 10.7) (Fig. 3). After the removal of one study [42] a similar magnitude of effect was observed (*moderate* SMD = 1.05, 95% CI 0.36–1.74, *p* = 0.007, *I*^2^ = 86%), making the overall estimate of the effect robust. The quality of evidence was downgraded from high to moderate due to the high heterogeneity of included studies (Table 3). Additionally, sub-analyses revealed that the effect of OIS was moderated by participants’ initial ferritin concentration (*Q* = 7.718; *p* = 0.005) and study duration (*Q* = 6.573; *p* = 0.010), but not by the daily average dose of elemental iron intake (*Q* = 1.139; *p* = 0.286) (Supplementary Table 2).

**Fig. 4:**
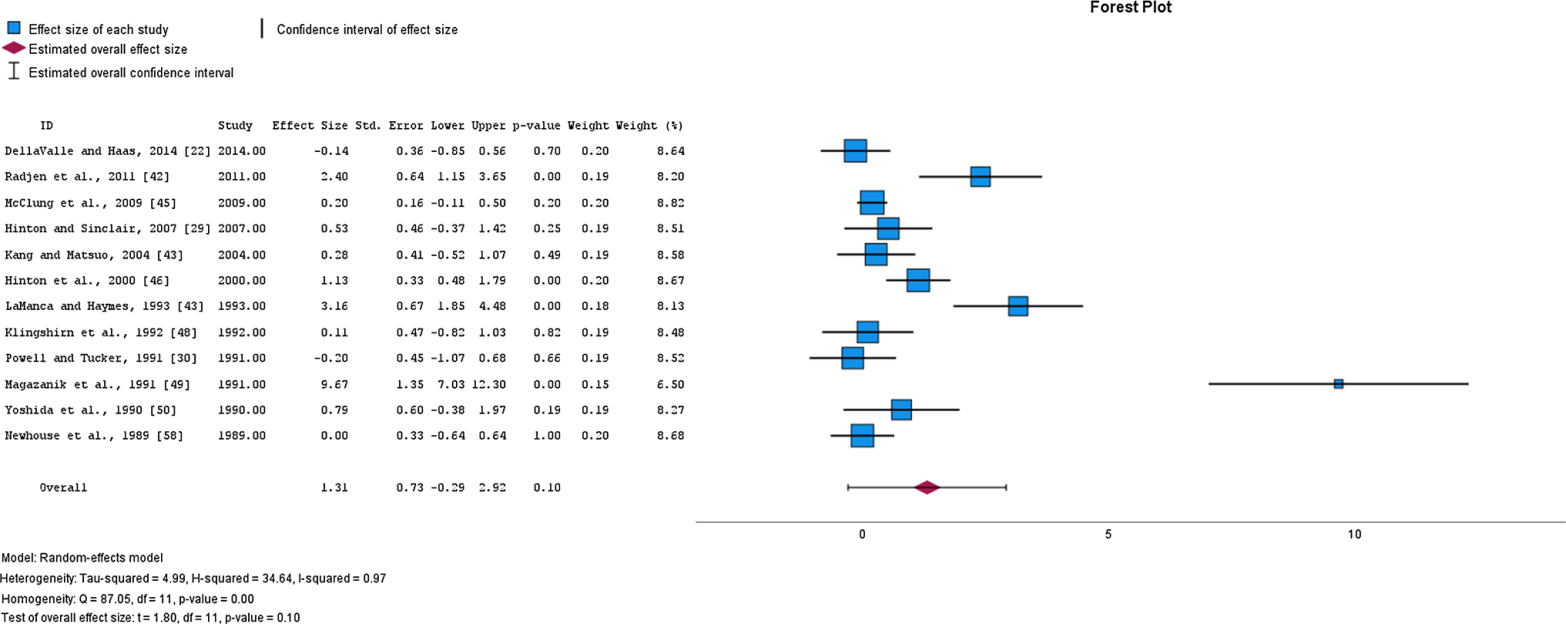
Changes in blood haemoglobin concentration after oral iron supplementation

Meta-analysis of 13 studies with a total of 435 participants demonstrated a *large*, yet non-significant increase of blood haemoglobin concentration after OIS (SMD = 1.31, 95% CI − 0.29 to 2.93, *p* = 0.099, *I*^2^ = 97%; EXP PRE mean ± SD = 133.7 ± 7.3, EXP POST = 134.9 ± 5.9; CON PRE = 134.0 ± 7.3; CON POST = 131.1 ± 6.8) (Fig. 4). After the removal of one study [49], a lower magnitude of effect was observed (*moderate* SMD = 0.65, 95% CI − 0.29 to 1.33, *p* = 0.059, *I*^2^ = 84%), making the overall estimate of the effect less robust (Supplementary Table 2). The quality of evidence was downgraded from high to moderate due to the high heterogeneity of included studies (Table 3). Additionally, sub-analyses revealed that the effects of intervention were not moderated by initial ferritin levels of participants, study duration, daily average dose of elemental iron intake, or initial haemoglobin levels (all *p* > 0.05) (Supplementary Table 2). Given all the above, the outlier effect observed in the study by Magazanik et al. [49] is unlikely to be ruled out by simply comparing the available data on iron status. However, these investigators solely included recreationally trained subjects (Tier 1) with lowest *V*O_2max_ values, who were more susceptible to experiencing improvements after longer training interventions (≥ 6 weeks).

**Fig. 5:**
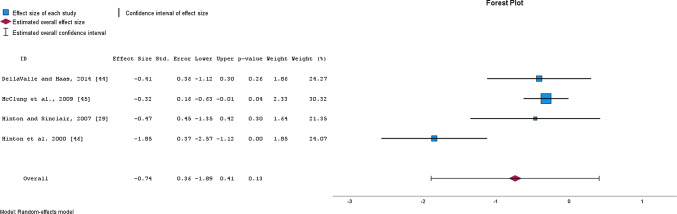
Changes in serum transferrin receptor concentration after oral iron supplementation

**Fig. 6:**
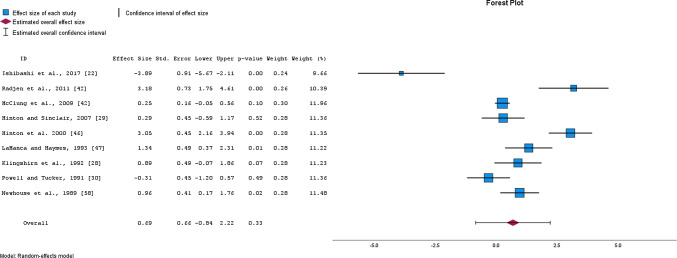
Changes in transferrin saturation after oral iron supplementation

OIS resulted in a *moderate*, yet non-significant decrease in serum transferrin receptor concentration as assessed from 4 studies with a total of 276 participants (SMD = − 0.74, 95% CI − 1.89 to 0.41, *p* = 0.133, *I*^2^ = 81%; EXP PRE mean ± SD = 5.6 ± 1.2, EXP POST = 5.0 ± 1.0; CON PRE = 5.7 ± 1.2; CON POST = 5.8 ± 1.3) (Fig. 5), whereas a significant *small* decrease was observed after the removal of one study [46] (SMD = − 0.35, 95% CI − 0.50 to − 0.19, *p* = 0.01, *I*^2^ = 0%). Moreover, a *moderate,* yet non-significant increase in transferrin saturation, as assessed from 9 studies with a total of 380 participants, was observed (SMD = 0.69, 95% CI − 0.84 to 2.22, *p* = 0.330, *I*^2^ = 95%; EXP PRE mean ± SD = 23.1 ± 7.9, EXP POST = 28.5 ± 9.6; CON PRE = 21.8 ± 7.4; CON POST = 22.9 ± 7.1) (Fig. 6). Removing one study [22] from the analysis resulted in a *moderate* increase in transferrin saturation (SMD = 1.14, 95% CI − 0.09 to 2.18, *p* = 0.036, *I*^2^ = 87%). The study by Ishibashi and colleagues [22], considered an outlier, was the sole report showing a noteworthy decrease in transferrin saturation levels in the experimental group compared to the control subjects. This observation can be linked to the accumulation of a substantial amount of running in a very short time—specifically, 100 km on average during three days only. Consequently, higher fatigue and systemic inflammation after training were indicated by elevated self-reported fatigue, muscle soreness, IL-6, CK, and CRP levels, respectively [22]. This was further confirmed by significantly higher hepcidin levels in the experimental group compared to control subjects after the 3-day training period (effect size = 0.90, *p* = 0.025). The quality of evidence for serum transferrin receptor concentration was downgraded from high to low (due to moderate to high heterogeneity and low sample size of the included studies). In contrast, the evidence for transferrin saturation was downgraded from high to moderate, due to the high heterogeneity of included studies only (Table 3).

#### Effect of Iron Supplementation on Maximal Oxygen Consumption

**Fig. 7:**
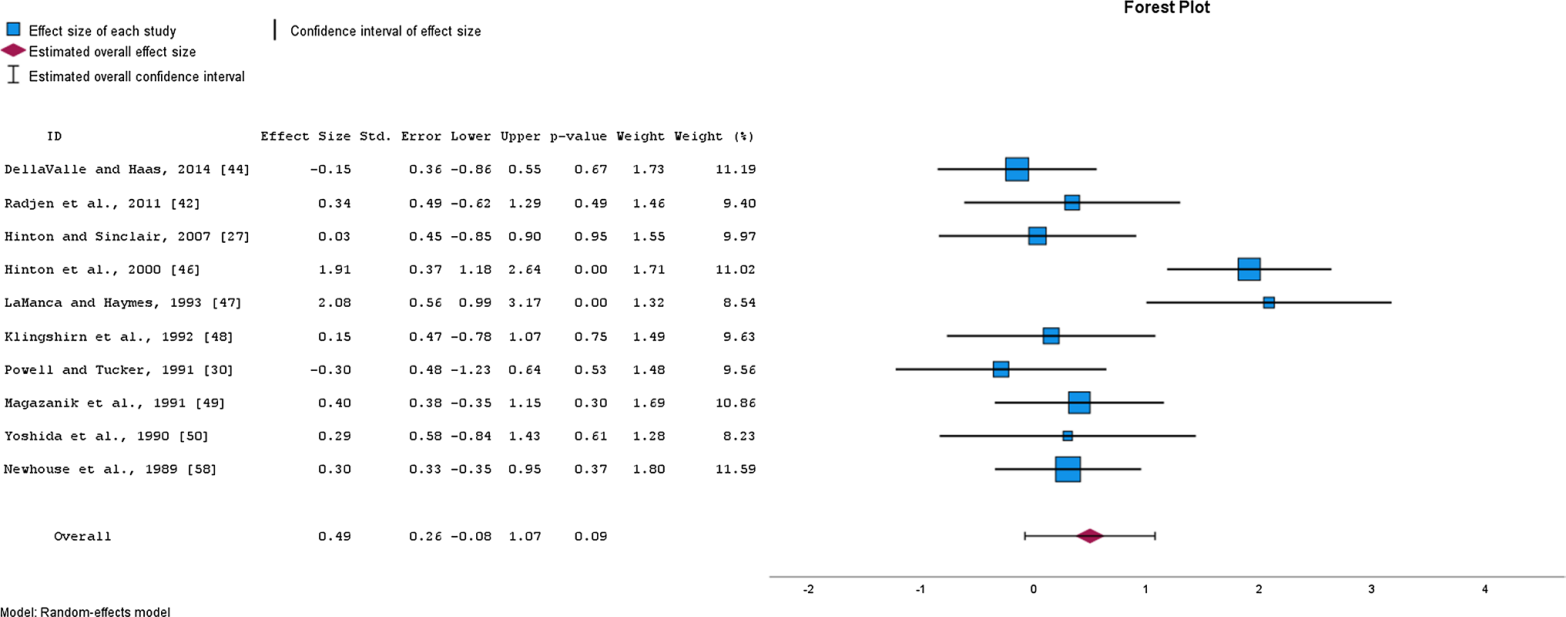
Changes in maximal oxygen consumption (*V*O_2max_) after oral iron supplementation

Meta-analysis of 8 studies with a total of 243 participants demonstrated a *small* positive, yet non-significant effect of iron supplementation on *V*O_2max_ (SMD = 0.49, 95% CI − 0.09 to 1.07, *p* = 0.086, *I*^2^ = 70%; EXP PRE mean ± SD = 47.4 ± 4.4, EXP POST = 49.5 ± 4.0; CON PRE = 46.9 ± 4.4; CON POST = 48.3 ± 4.3) (Fig. 7). In contrast, removing one study [47] from the analysis, revealed a *small*, yet non-significant negative effect on *V*O_2max_ (SMD = 0.35, 95% CI − 0.16 to 0.86, *p* = 0.152, *I*^2^ = 62%), making the overall estimate of the effect on *V*O_2max_ robust. The quality of evidence for *V*O_2max_ was downgraded from high to low, due to the high heterogeneity and low sample size of included studies (Table 3).

### Meta-Regression Analysis

**Fig. 8:**
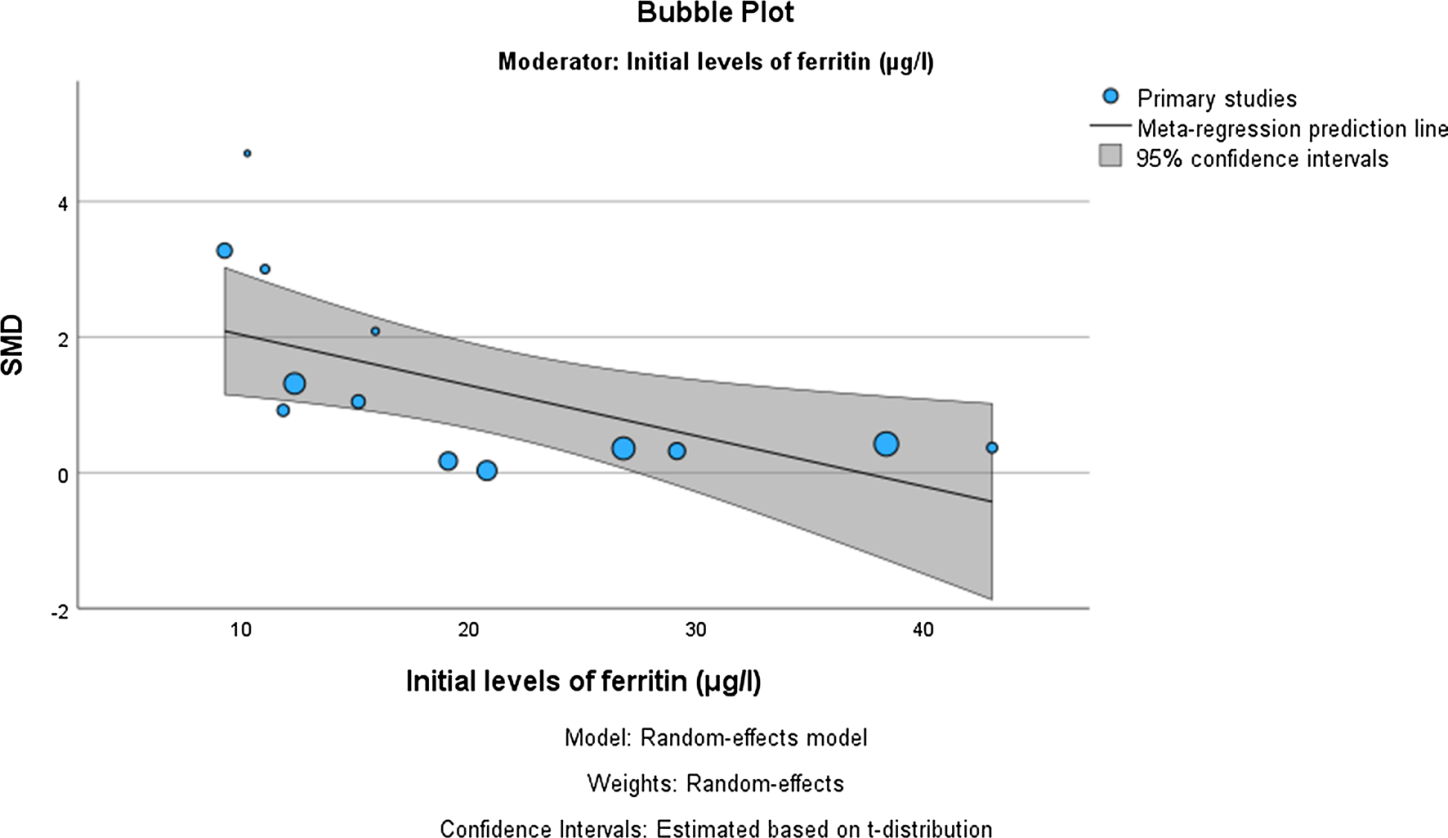
Meta-regression analysis predicting the effects of initial serum ferritin concentration on the effectiveness of oral iron supplementation on post-intervention ferritin levels, presented with a bubble plot. *SMD* standardized mean difference

Table 4 presents the results of meta-regression analysis, investigating whether the effects of OIS on *V*O_2max_, haemoglobin, and serum ferritin concentration were moderated by the initial concentration of ferritin, initial haemoglobin levels (only for haemoglobin), daily dose of elemental iron intake, age of participants, duration of supplementation, methodological quality of included studies (PEDro score) and initial levels of *V*O_2max_ (only for *V*O_2max_). The results demonstrate, that the effect of OIS on participants’ ferritin concentration depended upon the initial serum ferritin concentration (β = − 0.075, *p* = 0.023), with a negative association observed (the smaller the initial ferritin concentration, the larger the effect of OIS) (Fig. 8). No other significant associations were observed for the other parameters.

**Table 4:** Meta-regression analysis for different variables to predict the effects of oral iron supplementation on ferritin and haemoglobin levels

	Coefficient	Standard error	*t* value	*P* value	95% lower CI	95% upper CI
Ferritin						
Initial levels of serum ferritin (μg/l)	− 0.075	0.0284	− 2.629	0.023	− 0.137	− 0.012
Average dose of elemental iron intake (mg/day)	0.004	0.0083	0.460	0.655	− 0.015	0.022
Duration of intervention (days)	0.034	0.0209	10.604	0.137	− 0.012	0.080
Age of participants (years)	0.025	0.1001	0.249	0.808	− 0.195	0.245
Methodological quality of included studies (PEDro score)	− 0.088	0.3042	− 0.289	0.778	− 0.757	0.582
Haemoglobin						
Initial levels of serum ferritin (μg/l)	− 0.041	0.0871	− 0.468	0.649	− 0.235	0.153
Initial haemoglobin levels (g/l)	− 0.157	0.0980	− 1.602	0.140	− 0.375	0.061
Average dose of elemental iron intake (mg/day)	− 0.009	0.0210	− 0.431	0.677	− 0.057	0.039
Duration of intervention (days)	0.041	0.0561	0.739	0.477	− 0.083	0.166
Age of participants (years)	− 0.123	0.1843	− 0.665	0.521	− 0.533	0.288
Methodological quality of included studies (PEDro score)	0.273	0.5499	0.496	0.630	− 0.952	1.498
*V*O_2max_						
Initial levels of serum ferritin (μg/l)	− 0.070	0.0312	− 2.252	0.054	− 0.142	0.002
Initial haemoglobin levels (g/l)	− 0.043	0.0373	− 1.156	0.281	− 0.129	0.043
Average dose of elemental iron intake (mg/day)	− 0.002	0.0074	− 0.299	0.774	− 0.020	0.015
Duration of intervention (days)	0.017	0.0206	0.816	0.438	− 0.031	0.064
Age of participants (years)	0.018	0.0613	0.300	0.772	− 0.123	0.160
Methodological quality of included studies (PEDro score)	0.188	0.1786	1.052	0.324	− 0.224	0.600
Initial level of *V*O_2max_ (ml/kg·min)	− 0.054	0.0258	− 2.093	0.070	− 0.114	0.005

*VO*_*2max*_ maximal oxygen consumption, *CI* confidence interval

## Discussion

This systematic review and meta-analysis synthesized the available evidence on the effects of OIS on haematological and performance-related parameters in healthy adult athletes. The results demonstrated that OIS significantly increases serum ferritin concentration. Although increases in blood haemoglobin concentration were observed, those alterations were non-significant. Similarly, a non-significant decrease in serum transferrin concentration and moderate trend towards higher transferrin saturation were observed. For all these results, moderate quality of evidence was observed. Furthermore, after OIS, a small positive effect on *V*O_2max_ was observed in young healthy athletes, with low quality of evidence.

Iron status is regulated by various proteins, among which *ferritin* is an intracellular iron-binding protein and the primary storage of iron in the cells [51]. In steady-state conditions, the concentration of serum ferritin is correlated with iron stored in liver cells, ﻿which means measuring serum ferritin concentration enables the estimation of cellular iron stores [52]. However, the interpretation of serum ferritin concentration can be challenging due to its correlation with inflammation [52]. During periods of systemic inflammation, which are exacerbated by intensive and prolonged physical training [53], a decrease in iron status may be therefore difficult to detect [54], as ferritin concentration can show normal values despite depleted iron stores. Therefore, the use of serum ferritin concentration alone to assess iron status in such conditions may not be reliable [52–54] *Transferrin* is an iron-transporting protein found in blood plasma that transports iron from absorption sites to tissues, such as bone marrow and liver. Transferrin then enters cells through a receptor-mediated endocytosis and releases iron due to a lower pH of the endocytotic vesicle. One of the parameters associated with transferrin is *transferrin saturation* [55]. It is measured as a percentage and represents the ratio of serum iron concentration (i.e. a sum of serum transferrin and serum ferritin concentrations) to the total iron-binding capacity (i.e. a value reflecting full capacity of serum transferrin to bind iron). It has been reported that in cases of iron deficiency, plasma transferrin concentration increases and transferrin saturation decreases [56]. Our findings confirm previous reports [56] that iron status improves in athletes receiving OIS.

Physical activity-related inflammation can cause an increase in serum ferritin concentration, which may not accurately reflect cellular iron stores. Nevertheless, the results of this meta-analysis confirm that pre-supplementation serum ferritin concentration can, to a certain extent, indicate the success of OIS. In the present meta-analysis, we found that in studies where the highest pre-supplementation average serum ferritin concentration of the subjects was 12 µg/l or lower, an increase in serum ferritin concentration was observed after supplementation, with larger increases seen in those with lower pre-supplementation ferritin levels [24, 40–43].

It is worth noting that on the basis of their haemoglobin concentration (higher than 115 g/l), transferrin saturation (higher than 16%), and serum ferritin concentration (lower than 35 µg/l), these subjects would correspond to the first stage of iron deficiency according to Peeling et al.[5]. However, their low serum ferritin concentration of 12 µg/l or lower classifies them in the third stage of iron deficiency according to Peeling et al. [5], and thus in the iron deficiency anemia group.

In subjects with higher pre-supplementation serum ferritin concentration (those above 12 µg/l), only minimal, if any, increases in serum ferritin concentration after oral iron supplementation were observed [18, 22, 30, 43, 45]. Thus, although the present meta-analysis has demonstrated that oral iron supplementation leads to a significant increase in iron stores, as indicated by an increase in serum ferritin concentrations, it seems that the magnitude of this increase is not sufficient to be clinically meaningful, i.e. it may not be high enough to raise iron stores to a level, at which an athlete would not experience health- and/or performance-related consequences of iron deficiency. More specifically, even though oral iron supplementation resulted in a significant increase in serum ferritin concentration, it remained below 35 µg/l in 10 out of 13 studies after supplementation, and thus below the limit for the first stage of iron deficiency [5].

The doses of OIS which induced a beneficial effect on haematological parameters differed from 16 [57] to 100 mg [47, 58] of elementary iron daily in the period between 6 [29, 40, 57] to 8 weeks [47, 58]. Studies which used shorter supplementation protocols [22, 30, 43] reported no or only negligible effects on serum ferritin or haemoglobin concentration in the present meta-analysis. It is worth noting that current treatment strategies [2] suggest OIS for IDNA (where ferritin is < 35 µg/l with normal haemoglobin levels) for the period of 4–12 weeks. In light of our findings, the optimal duration of OIS should be at least 6 to 8 weeks to observe a significant increase in ferritin levels. However, even in this case, the question remains regarding the magnitude of the supplementation effect. If ferritin levels are not expected to exceed 35 µg/L after 6–8 weeks of OIS, we should find a better way to assist and treat athletes with IDNA, as OIS does not seem to be an efficient way to replenish the depleted iron stores. We believe that a new clinical pathway of starting intravenous iron supplementation earlier should be considered. By implementing this strategy, the treating physician would assess whether there is a need for intravenous iron supplementation, and athletes would receive timely and efficient medical care to reduce the negative consequences of iron deficiency.

The decreased serum transferrin concentration after OIS observed in the present meta-analysis is in line with the findings of Chatard et al. [56]. The latter study found that in cases of iron deficiency, plasma transferrin concentration increases and transferrin saturation decreases. Although post-supplementation increase in transferrin saturation was only detected as a trend in the present meta-analysis, the observed effect was moderate. Thus, it is reasonable to conclude that OIS successfully reverses the physiological condition of iron deficiency in IDNA athletes. A decrease in serum transferrin concentration following OIS may be interpreted as a down-regulation of transferrin (following a lower absorption in enterocytes), when iron body stores are replenished.

The present meta-analysis revealed a small positive effect of OIS on maximal oxygen consumption. However, the latter result must be treated with caution. More specifically, if one were to study the potential ergogenic effects of OIS per se, the training regimen should remain the same throughout the supplementation period to ensure that performance parameters would not change due to training only. However, as OIS may improve training capacity, a steady-state training regimen is likely difficult to maintain and any increase in training load may consequently result in higher performance. In this case, it would be erroneous to speak about ergogenic effects of OIS per se, as it was the optimisation of training (through the reduction of negative effects of IDNA) that caused the effect.

According to the Position of the Academy of Nutrition and Dietetics, Dietitians of Canada, and the American College of Sports Medicine on Nutrition and Athletic Performance [4], routine, unmonitored iron supplementation is not recommended without clinical evidence of iron depletion. As oral iron supplementation can cause significant gastrointestinal side effects [27] and as its ergogenic effects have not been unequivocally proven in iron-deficient individuals, let alone in healthy non-anaemic individuals, it is even more sensible that professionals are included in any advice regarding iron supplementation.

### Limitations and Future Directions

The scoring of studies in meta-analyses is usually performed by a team of independent experts, yet, in the present study, the majority of scoring was performed by the first author. Nevertheless, we estimate that the scoring was done correctly, because whenever any doubts were raised, the first author consulted with other authors to reach the final agreement. Furthermore, although the duration of OIS was shorter than in the other studies included in the present meta-analysis, and the daily iron intake was also low, the study of Ishibashi et al. [22] was retained in our meta-analysis, because the length of OIS was not adopted as one of the inclusion/exclusion criteria for the meta-analysis. In addition, in the majority of studies included in the present meta-analysis, the state of inflammatory response was not defined, so this variable could not be included in the meta-analysis. Nevertheless, it should be stressed that the state of inflammatory response is an important factor for the interpretation of iron status and the effect of iron supplementation, as physical activity triggers an inflammatory response, which largely determines the response to OIS. Similarly, in most of the studies included in the present meta-analysis, the authors did not assess the total daily dietary iron intake, which is crucial for the introduction and evaluation of any nutritional intervention. Therefore, it would be highly desirable to include these data in any future research in this area. Finally, the number of studies included in the present meta-analysis was quite small, which indicates the need for additional research work in this area. We also allow for the possibility that our search criteria may not have identified all the published peer-reviewed papers on this research topic, either due to publications in a non-English language or due to some other factor. Nevertheless, we believe that the present meta-analysis covered most of the research to date in the investigated area.

## Conclusion

The results of the present literature review and meta-analysis confirm the plausibility of OIS in IDNA athletes, while presenting no beneficial effects of OIS on haematological or performance-related parameters in athletes with normal serum ferritin levels. Additionally, we have highlighted some issues that should be considered when a decision is made about the treatment of IDNA athletes. The physiological requirements of modern sport are very high. Sports experts who deal with athletes often increase the amount of high-intensity training with the ultimate goal of boosting their working capacity and competitiveness. This can result in a disturbed physiological state, where iron demands are elevated. If the balance between high metabolic stress and proper recovery strategies is not met, this can lead to a decrement in athletes’ health and performance. With current treatment strategies, some athletes may take oral iron supplementation for some time but end up with ferritin levels that still do not meet their needs. Thus, dietitians and physicians should work together to optimize an oral iron supplementation treatment approach that would be safe and effective for athletes, considering each athlete individually.

### Supplementary Information

Below is the link to the electronic supplementary material.

Supplementary file1 (DOCX 30 kb)

## Declarations

### Data Availability

Data will be provided upon reasonable request to the corresponding author.
