# Supplementary material for: Effects of Oral Iron Supplementation on Blood Iron Status in Athletes: A Systematic Review, Meta-Analysis and Meta-Regression of Randomized Controlled Trials
Source: Sports Med. 2024 Feb 26;54(5):1231–47. doi: 10.1007/s40279-024-01992-8 (PMC11127818; doi:10.1007/s40279-024-01992-8)
Supplement: Supplementary file 1 — Supplementary file1 (DOCX 30 kb) [file 40279_2024_1992_MOESM1_ESM.docx]

**Supplementary table 1.** Additional characteristics of studies included in the meta-analysis with focus on the interventions.

| Study | Exercise intervention during supplementation | Method used to monitor load | Exercise intervention during supplementation |
| --- | --- | --- | --- |
| Ishibashi et al. 2017 [22] | All subjects completed the six aerobic exercise sessions at 75% of the VO2max | Heart rate, running velocity. | Yes |
| DellaValle and Haas 2014 [44] | Training included general aerobic conditioning (cycling, running, and rowing on ergometer), resistance training, and high intensity on-water rowing (as weather permitted) | Session RPE. | Yes |
| Radjen et al. 2011 [42] | Participants maintained their pre-supplement exercise training schedule (volleyball training and competition) | Not reported | No |
| McClung et al. 2009 [45] | Participant were included in basic combat training: 4–6 d/week, 60-90 minutes of aerobic and strength training. | Not reported. | Yes |
| Hinton and Sinclair 2007 [29] | Subjects trained on ergometer for 30 min/day, 5 days/wk at 75–85% of maximum heart rate. The training sessions included a 4-min warm-up followed by a 25-min cycling session divided between workloads that allowed subjects to achieve a target HR of 75% of maximum HR (HRmax) and 85% HRmax. Over the course of the 4-wk training program, the time spent at 75% HRmax was decreased from 20 to 10 min, with a corresponding increase in the duration of cycling at 85% HRmax. | Heart rate, average cadence, and work (in W) for each training session. | Yes |
| Kang and Matsuo 2004 [43] | The subjects severely trained for 7-9 hours every-day. There were 2 soccer training sessions and 1 weight training session per day. | Not reported. | No |
| Hinton et al. 2000 [46] | Participants maintained their pre-supplement exercise training schedule (60 min aerobic exercise, 3 days/week) | Daily exercise log (exercise time, mode, pace and intensity). | No |
| LaManca and Haymes 1993 [47] | Participants maintained their pre-supplement exercise training schedule (running was the major form of exercise, 4.8 – 11.2 km/day, 3-5 days/week, average running pace 13.7 km/h) | Not reported. | No |
| Klingshirn et al. 1992 [48] | Participants maintained their pre-supplement exercise training schedule (at least 120 min aerobic exercise, 3 days/week | Daily exercise log (number of miles run each day). | No |
| Powell and Tucker 1991 [30] | Participants maintained their pre-supplement exercise training schedule (aerobic exercise, cross- country track 65 km – 80 km/week) | Not reported. | No |
| Magazanik et al. 1991 [49] | The training consisted each day of 5-6 h of intensive physical activity, 6 days a week, and included field running (4-5 km a day), jumping, weightlifting and cliff climbing. | Not reported. | Yes |
| Yoshida et al. 1990 [50] | Participants maintained their pre-supplement exercise training schedule (aerobic exericse including countinous and interval running), 6 days/week, 3 hours/day, 32-48 km of running per day) | Training log supervised by coach | No |
| Newhouse et al. 1989 [58] | Participants maintained their pre-supplement exercise training schedule (at least 120 min aerobic exercise, 3 days/week). | Not reported. | No. |

**Supplementary table 2.** Effects of oral iron supplementation on haematological and performance-related parameters, relative to different grouping variables.

| Independent variables | SMD | SE | *t value* | *P value* | 95 % CI | | *I^2^* (%) | *df* | *Q value and (p) between groups* |
| --- | --- | --- | --- | --- | --- | --- | --- | --- | --- |
| Ferritin | | | | | | | | | |
| All studies | 1.270 | .3804 | 3.338 | .006 | .441 | 2.099 | 90 | 12 | NA |
| One study excluded | 1.046 | .3131 | 3.342 | .007 | .357 | 1.736 | 86 | 11 |  |
| *Sub-analysis on initial serum ferritin levels* | | | | | | | | | |
| ≤12 μg/l | 2.484 | .7000 | 3.548 | .024 | .540 | 4.427 | 86 | 4 | 7.718 (0.005) |
| >12 μg/l | .519 | .1911 | 2.717 | .030 | .067 | .971 | 39 | 7 |  |
| *Sub-analysis on study duration* | | | | | | | | | |
| <6 weeks | .269 | .0601 | 4.475 | .046 | .010 | .528 | 0 | 2 | 6.573(0.010) |
| ≥6 weeks | 1.591 | .4595 | 3.462 | .007 | .551 | 2.630 | 92 | 7 |  |
| *Sub-analysis on daily average dose of elemental iron intake* | | | | | | | | | |
| <60 mg | .788 | .4209 | 1.873 | .110 | -.242 | 1.818 | 89 | 6 | 1.139 (0.286) |
| ≥60 mg | 1.437 | .4553 | 3.157 | .034 | .173 | 2.702 | 73 | 4 |  |
| Haemoglobin | | | | | | | | | |
| All studies | 1.313 | .7286 | 1.801 | .099 | -.291 | 2.916 | 97 | 11 | NA |
| One study excluded | .651 | .3051 | 2.134 | .059 | -.029 | 1.331 | 84 | 10 |  |
| *Sub-analysis on initial serum ferritin levels* | | | | | | | | | |
| ≤12 μg/l | 1.389 | .5601 | 2.480 | .068 | -.166 | 2.944 | 84 | 4 | 0.001 (0.976) |
| >12 μg/l | 1.349 | 1.2933 | 1.043 | .337 | -1.816 | 4.514 | 99 | 6 |  |
| *Sub-analysis on study duration* | | | | | | | | | |
| <6 weeks | .065 | .2358 | .275 | .829 | -2.931 | 3.061 | 0 | 1 | *3.1854 (0.074)* |
| ≥6 weeks | 1.608 | .8721 | 1.844 | .098 | -.365 | 3.581 | 98 | 9 |  |
| *Sub-analysis on daily average dose of elemental iron intake* | | | | | | | | | |
| <60 mg | 1.780 | 1.4863 | 1.198 | .285 | -2.041 | 5.601 | 99 | 5 | 0.495 (0.482) |
| ≥60 mg | .699 | .5992 | 1.167 | .308 | -.964 | 2.363 | 86 | 4 |  |
| *Sub-analysis on initial haemoglobin levels* | | | | | | | | | |
| <134 | 1.916 | 1.1011 | 1.740 | .125 | -.687 | 4.520 | 98 | 7 | 2.420 (0.120) |
| >=134 | .249 | .2104 | 1.185 | .321 | -.420 | .919 | 0 | 3 |  |
| Transferrin saturation | | | | | | | | | |
| All studies | .688 | .6633 | 1.037 | .330 | -.841 | 2.218 | 95 | 8 | NA |
| One study excluded | 1.144 | .4434 | 2.580 | .036 | .095 | 2.192 | 89 | 7 |  |
| *Sub-analysis on initial serum ferritin levels* | | | | | | | | | |
| ≤12 μg/l | 1.703 | .5783 | 2.945 | .042 | .097 | 3.309 | 84 | 4 |  |
| >12 μg/l | -.620 | 1.0374 | -.598 | .592 | -3.922 | 2.681 | 96 | 3 | 4.066 (0.044) |
| *Sub-analysis on study duration* | | | | | | | | | |
| <6 weeks | -2.016 | 1.7869 | -1.128 | .462 | -24.720 | 20.689 | 92 | 1 | *3.338 (0.068)* |
| ≥6 weeks | 1.348 | .4509 | 2.991 | .024 | .245 | 2.452 | 95 | 6 |  |
| *Sub-analysis on daily average dose of elemental iron intake* | | | | | | | | | |
| <60 mg | 0.00 | 1.41 | -0.001 | 1 | -4.48 | 4.48 | 98 | 3 | 0.247 (0.619) |
| ≥60 mg | 0.71 | 0.36 | 1.979 | 0.142 | -0.43 | 1.85 | 59 | 3 |  |
| Transferrin Receptor Concentration | | | | | | | | | |
| All studies | -0.74 | 0.36 | -2.05 | 0.133 | -1.89 | 0.41 | 81 | 3 | NA |
| One study excluded | -0.35 | 0.04 | -9.758 | 0.01 | -0.50 | -0.19 | 0 | 2 |  |
| VO_2_max | | | | | | | | | |
| All studies | .493 | .2552 | 1.931 | .086 | -.085 | 1.070 | 70 | 9 | NA |
| One study excluded | .349 | .2207 | 1.583 | .152 | -.160 | .858 | 62 | 8 |  |
| *Sub-analysis on initial serum ferritin levels* | | | | | | | | | |
| ≤12 μg/l | .897 | .4491 | 1.998 | .116 | -.350 | .897 | 79 | 4 | 2.562 (0.109) |
| >12 μg/l | .123 | .1347 | .913 | .413 | -.251 | .123 | 0 | 4 |  |
| *Sub-analysis on daily average dose of elemental iron intake* | | | | | | | | | |
| <60 mg | .551 | .4709 | 1.171 | .326 | -.947 | 2.050 | 83 |  | 0.018 (0.894) |
| ≥60 mg | .470 | .3942 | 1.193 | .299 | -.624 | 1.565 | 69 |  |  |
| SMD - standardized mean difference; SE – standard error; 95 % CI - 95% confidence interval; I^2^ (%) – study heterogeneity in percentage; df – degrees of freedom | | | | | | | | | |
